# Fabrication of Superhydrophobic Ti–6Al–4V Surfaces with Single-Scale Micotextures by using Two-Step Laser Irradiation and Silanization

**DOI:** 10.3390/ma13173816

**Published:** 2020-08-29

**Authors:** Haidong He, Risheng Hua, Xuan Li, Chunju Wang, Xuezhong Ning, Lining Sun

**Affiliations:** 1 College of Mechanical and Electrical Engineering, Soochow University, Suzhou 215006, China; hdhe@suda.edu.cn (H.H.); 20195229040@stu.suda.edu.cn (R.H.); lnsun@suda.edu.cn (L.S.); 2 Robotics and Microsystems Center, Soochow University, Suzhou 215006, China; 3 School of Materials Science and Engineering, Harbin Institute of Technology, Harbin 150001, China; 4 TronLight Precision Engineering Technology, Ltd., Suzhou 215006, China; 21425050@zju.edu.cn

**Keywords:** laser irradiation, microtextures, surface modification, superhydrophobicity, functional surface, titanium alloys

## Abstract

Laser irradiation is a popular method to produce microtextures on metal surfaces. However, the common laser-produced microtextures were hierarchical (multiscale), which may limit their applicability. In this paper, a method of two-step laser irradiation, combining first-step strong ablation and sequentially second-step gentle ablation, was presented to produce micron-rough surface with single-scale microtextures. The effect of laser fluence on the Ti–6Al–4V surface morphology and wettability were investigated in detail. The morphology results revealed that the microtextures produced using this method gradually evolved from multiscale to single-scale meanwhile from microprotrusions to microholes with increasing the second-step laser fluence from 0.0 to 2.4 J/cm^2^. The wettability and EDS/XPS results indicated that attributing to the rich TiO_2_ content and micron roughness produced by laser irradiation, all the two-step laser-irradiated surfaces exhibited superhydrophilicity. In addition, after silanization, all these superhydrophilic surfaces immediately turned to be superhydrophobic with close water contact angles of 155–162°. However, due to the absence of nanotextures, the water-rolling angle on the superhydrophobic surfaces with single-scale microtextures distinctly larger than those with multiscale ones. Finally, using the two-step laser-irradiation method and assisted with silanization, multifunctional superhydrophobic Ti–6Al–4V surfaces were achieved, including self-cleaning, guiding of the water-rolling direction and anisotropic water-rolling angles (like the rice-leaf), etc.

## 1. Introduction

Superhydrophobic metal surfaces with a water contact angle (WCA) larger than 150° have attracted great interest in both academic research and industrial applications [1,2,3]. Recently, it was found that controlling the wettability of the metallic biomaterial surfaces, such as titanium and its alloys, to be superhydrophobic could effectively enhance their hemocompatibility [4,5]. This was quite promising to be applied in the blood-contacting implants for reducing and even preventing the problems of hemolysis, coagulation and formation of thrombus. In addition, the superhydrophobic surface could also effectively reduce the bacterial adhesion, which was beneficial for preventing bacterial infection in the clinical treatment [6]. It was important to note that the aforementioned two factors of good hemocompatibility and antibacterial property were urgently desired by the biomaterials used in the interventional therapy.

It is well known that microtextures and low surface energy are two main factors for achieving superhydrophobicity [7]. Among the various micromachining techniques, laser irradiation is a popular method to produce microtextures on the metal surfaces, due to its advantages of location flexible, environment friendly, wide processing range and time-efficiency [8,9]. After reducing the surface energy (e.g., using chemical modification with silanization), the micron-rough surfaces produced by laser irradiation generally exhibited superhydrophobicity. For example, Li et al. [10] produced superhydrophobic Ti surface with the WCA of 156.9° and water-rolling angle (WRA) of 4.7° by using femtosecond laser irradiation. Chen et al. [11] produced a reed-leaf-like (superhydrophobic stainless surface with the WCA of 157 ± 1° and WRA of 1.0 ± 0.5° by using nanosecond laser irradiation, and it exhibited good corrosion resistance. Yang et al. [12] found that the laser-fabricated aluminum superhydrophobic surface with the WCA of 160.6 ± 1.5° and WRA of 3 ± 1° exhibited perfect self-cleaning capability, long-term stability, and excellent chemical stability in acidic as well as alkaline solutions. Fadeeva et al. [13] reported that the laser-produced superhydrophobic Ti surface with the WCA of 166 ± 4° exhibited good antibacterial property. Xin et al. [14] reported that superhydrophobic Ti–6Al–4V surface with grid-shape multiscale microtextures produced by laser irradiation exhibited good drag-resistance. However, in most of the previous investigations, the laser-produced microtextures for achieving superhydrophobic surfaces were commonly multiscale, attributing to the laser strong ablation of target material. The type of the produced microtextures was generally similar to microprotrusion. In addition, the obtained superhydrophobic surfaces were generally with a low WRA less than 5°. These inevitable characteristics may reduce the flexibility and limit the applicability of laser irradiation technology.

In this paper, a novel but simple method of two-step laser irradiation is presented, in which the first-step laser process (strong ablation) was used to produce micron-rough surface while the second-step one (gentle ablation) was applied to modify the characteristics of the microtextures produced in the first-step laser process. The effect of laser fluence on the morphology, roughness, chemical composition as well as wettability of Ti–6Al–4V surfaces was investigated and discussed in detail. Using this method, the morphology of the microtextures could be easily modified from multiscale to single-scale as well as from microprotrusions to microholes by simply adjusting the laser fluence applied in the second-step laser process. In addition, it was confirmed that through applying the second-step laser process in a selective region, superhydrophobic surfaces with various functions could be achieved, including self-cleaning, water-droplet selective rolling in different area, relative high adhesion, guiding of the water-rolling direction, etc. The results imply the potential applications of these surfaces in a variety of fields, such as biomedical, microfluidic and so on.

## 2. Materials and Methods

### 2.1. Principle

**Figure 1:**
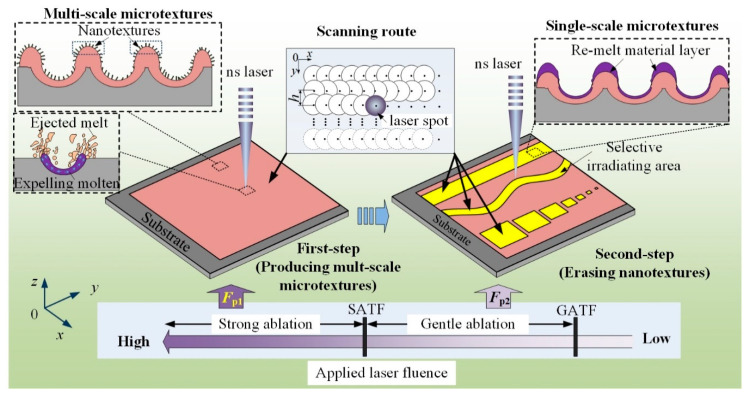
Principle of two-step laser-irradiation method to achieve micron-rough surface with single-scale microtextures.

Figure 1 presents the principle of two-step laser-irradiation method. To facilitate the narrative, subscript “1” was added to the symbol of the laser irradiation parameters involved in the first-step process (e.g., laser fluence *F*_p1_, scanning speed *v*_1_ and hatching distance *h*_1_), while subscript “2” was added to those in the second-step process (e.g., *F*_p2_, *v*_2_ and *h*_2_). In the first-step laser-irradiation process, high laser fluence (*F*_p1_) that above the strong ablation-threshold fluence (SATF) of the target material was applied, aiming to obtain a micron-rough surface. According to the mechanism of laser ablating solid material, at the condition of laser strong ablation (LSA), complex and violent physical processes occurred in the laser-material interface, resulting in the formation of abundant hierarchical/multiscale microtextures [15,16]. Different from that of LSA, at the condition of laser gentle ablation (LGA), soft physical processes mainly including melting and re-solidifying occurred in the laser-material interface and thus flat/smooth surface with small roughness was achieved. Actually, the basic mechanism of laser polishing technology was just LGA of surface asperity [17]. Therefore, if performing a second-step laser-irradiation process with *F*_p2_ between gentle ablation-threshold fluence (GATF) and SATF, a layer of material on the pre-fabricated multiscale microtextures would be melt, and thus the nanoscale characteristics (nanotextures) on them were removed. During the second-step laser process—because the thickness of the re-melt material layer was thin—the surface roughness of the target substrate maintained to be in micron level [17]. As consequence, the micron-rough surface with single-scale microtextures was obtained. It was important to note that the position, shape as well as size of the laser-irradiated area in the second-step process (yellow regions in Figure 1) could be designed to be various for achieving different functional surfaces.

### 2.2. Pretreatment of Samples

The commercial Ti–6Al–4V plates (Dongguan city Yuhao metal materials Co., Ltd., Dongguan, China) with the dimension of 10 mm × 10 mm × 2 mm were used in this paper, the nominal compositions of which are shown in Table 1. Prior to laser processing, the plates were mechanically polished using SiC papers with various roughness ranging from P400 to P1500, successively. Then, the polished plates were ultrasonically cleaned in acetone, alcohol as well as deionized water for 5 min sequentially, following dried with pressurized air at room temperature. After performing these processes, the plates were called as the as-received substrates.

### 2.3. Laser Processing

An ultraviolet nanosecond laser source with the wavelength (*λ*) of 355 nm, pulse width (*τ*) of 10 ns and repetition rate (*f*) of 30 kHz was used in the laser irradiation system. A two mirror galvanometric scanner (GS) with a 160-mm-focal-length F-Theta objective lens (FTOL) was employed to accurately control the laser beam scanning in the two-dimensional plane. After focused by the FTOL, the diameter of the laser beam at the focal plane was about 20 μm in diameter (Waist radius, ω_0_ = 10 μm). The scanning route of the focused laser beam was consisted of multi parallel lines with a certain hatching distance (*h*). The scanning speed (*v*), the moving velocity of laser spot in the focal plane, was controlled by the GS. The more detailed expatiation of the laser irradiation system could be seen in our previous work [15]. Before performing each laser process, the average laser output power (*P*_a_) was measured by using a pyroelectric detector. Consequently, the laser pulse fluence (*F*_p_), defined as the ratio of laser pulse energy (*E*_p_) and the laser-spot acreage, could be obtained by [15]

(1) $$F_{p}=\frac{E_{p}}{\pi \omega_{0}^{2}}=\frac{E_{a}}{\pi \omega_{0}^{2}f}$$

All the laser process was performed in an ambient atmosphere.

### 2.4. Silanization

To obtain hydrophobic surface, the surface energy of the as-received and laser-irradiated substrates was reduced via modifying fluoro alkylsilane (FAS, 1H, 1H, 2H, 2H-perfluorooctyltriethoxysilane). The related process was mainly consisted of three steps, as follows [16]:

(1) First, the substrates were immersed into FAS ethanolic solution with a concentration of 2% (*v*/*v*) for 30 min in a water bath tank with constant temperature of 60 ± 1 °C
(2) Then, the substrates were baked in an oven at 120 ± 1 °C for one hour.
(3) Finally, the samples were self-cooling in the ambient atmosphere and then cleaned by the deionized water.

### 2.5. Characterization of Surface Properties

A scanning electron microscope (SEM; HSM-IT100, JEOL, Tokyo, Japan) was used to characterize the surface morphology of the samples. Energy dispersive spectroscopy (EDS; HSM-IT100, JEOL, Tokyo, Japan) and X-ray photoelectron spectroscopy (XPS; ESCALAB 250 XI, Thermo Fisher Scientific, Waltham, MA, USA) were used to examine the surface chemical compositions. The XPS spectra was referenced to the C1 s peak of aliphatic carbon at a binding energy of 284.8 eV. The roughness factors of arithmetic mean surface roughness *R*_a_ and the mean peak-to-valley height *R*_z_ were obtained by using a surface roughometer (Perthometer M1, Mahr GmbH, Esslingen, Germany) [18]. The WCA and WRA of the Ti–6Al–4V surfaces were characterized by using a sessile droplet method. The results of surface roughness, WCA as well as WRA were corresponded to an average of five measurements and are presented as mean ± standard deviation.

## 3. Results and Discussion

### 3.1. Surface Morphology and Roughness

#### 3.1.1. The Effect of First-Step Laser Fluence 

Based on the principle of the two-step laser-irradiation method presented in the Section 2.1, it was indispensable to find the SATF of Ti–6Al–4V material. Thus, in this section the effect of *F*_p1_ on the laser-irradiated surface morphology was investigated, first. According to the results of our previous study on laser-texturing pure titanium (Grade 2, TA2) [15], the scanning speed and hatching distance were selected to be constant of 42 mm/s and 1.0 μm, respectively.

**Figure 2:**
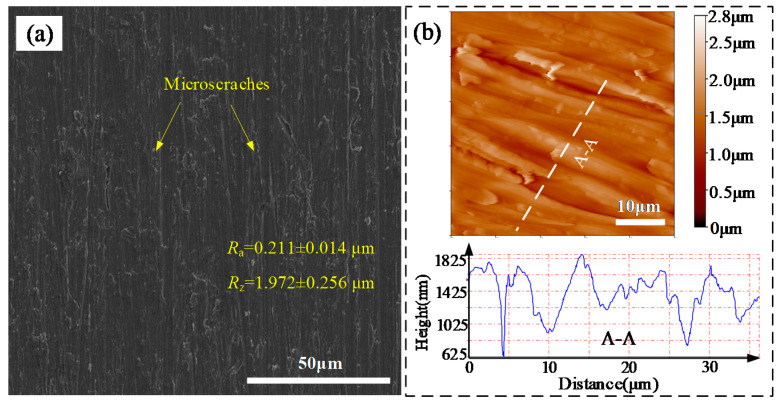
(**a**) SEM and (**b**) AFM morphologies of the as-received Ti–6Al–4V surface.

Figure 2 presents the morphologies of the as-received Ti–6Al–4V surface captured by SEM and AFM. From figure, it was clearly seen that a few microscratches (MS) with sharp profiles were formed on the Ti–6Al–4V surface after polished by a series of SiC papers. The average *R*_a_ and *R*_z_ of the as-received surface in the measuring length of 4.0 mm were about 0.211 ± 0.014 μm and 1.972 ± 0.256 μm, respectively. Therefore, the as-received Ti–6Al–4V surface was relative flat/smooth [19].

**Figure 3:**
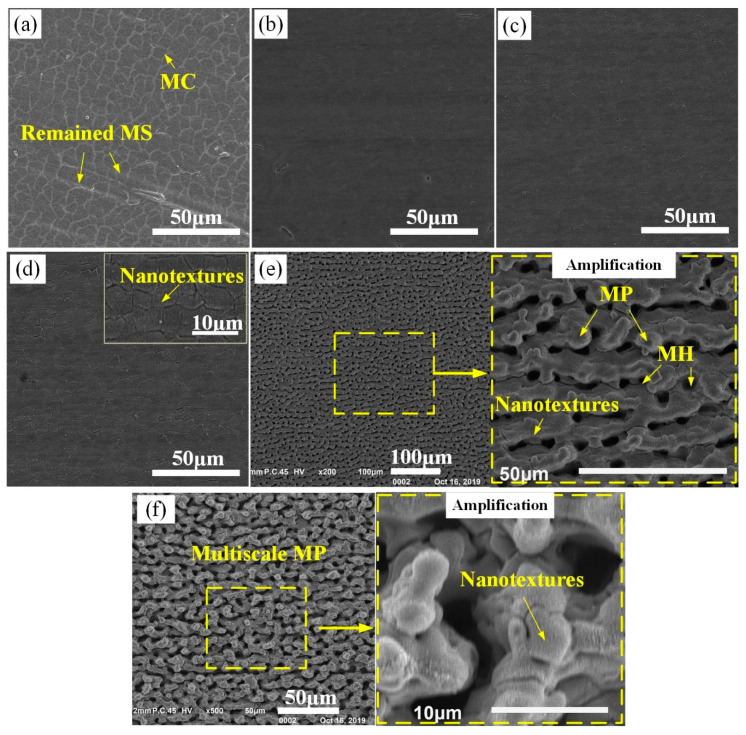
Morphology of first-step laser-irradiated surfaces at various *F*p1. (**a**) 1.1 J/cm^2^; (**b**) 1.9 J/cm^2^; (**c**) 2.4 J/cm^2^; (**d**) 2.9 J/cm^2^; (**e**) 3.4 J/cm^2^; (**f**) 4.0 J/cm^2^.

**Figure 4:**
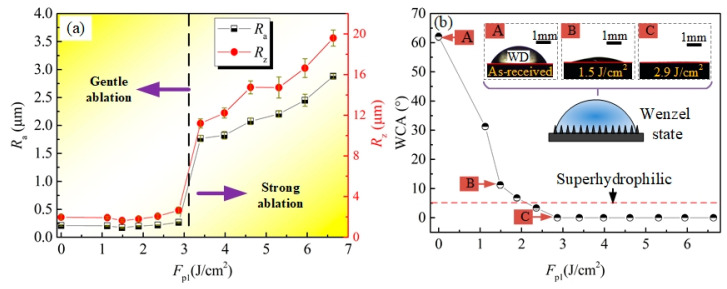
Effect of *F*p1 on the (**a**) surface roughness and (**b**) WCA of the Ti–6Al–4V surfaces without silanization.

Figure 3a–d shows that as applying *F*_p1_ ranging from 1.1 to 2.9 J/cm^2^, the Ti–6Al–4V surfaces were still flat just with some microcracks (MC) after laser irradiation. According to previous studies, the MC noticeable on these surfaces should be attributed to the repeated and fast melting/cooling during the laser-scanning irradiation process [10]. At the *F*_p1_ of 1.1 J/cm^2^ (Figure 3a), except for the distinct MC, the original MS were still observed in some region of the laser-irradiated surface. It indicated that the laser melting layer at the *F*_p1_ of 1.1 J/cm^2^ was quite thin, and thus some of the relative high MS on the as-received surface were not be removed completely. With increasing *F*_p1_ to 1.9 (Figure 3b) and 2.4 J/cm^2^ (Figure 3c), the laser-irradiated surfaces seemly changed to be smoother, meanwhile less MC were observed in them. In addition, the profile of original MS almost disappeared in these two surfaces. However, at the *F*_p1_ of 2.9 J/cm^2^, much MC were reappeared and nanotextures were observed in the laser-irradiated surface (Figure 3d). Note that, when increasing *F*_p1_ to 3.4 J/cm^2^, distinct rough surface with dense sunk-raised microtextures (microholes (MH) in the size of about 5–10 μm accompanied by microprotrusions (MP) covered with some nanotextures) was obtained, as shown in Figure 3e. Further increasing *F*_p1_ to 4.0 J/cm^2^, typical multiscale microtextures that MP covered with dense nanotextures were produced (Figure 3f). Figure 4a presents the evolution of the Ti–6Al–4V surface *R*_a_ and *R*_z_ with increasing *F*_p1_. It indicated that as applying *F*_p1_ ranging from 1.1 to 2.9 J/cm^2^, the change of Ti–6Al–4V surface roughness (*R*_a_ = 0.172–0.266 μm and *R*_z_ = 1.641–2.649 μm) was slight, which was just close to that of the as-received surface (*R*_a_ = 0.211 μm and *R*_z_ = 1.972 μm). However, note that, sharp increase of surface roughness to *R*_a_ = 1.767 μm and *R*_z_ = 11.199 μm occurred at the *F*_p1_ = 3.4 J/cm^2^, and then the surface roughness gradually increased with further increase of *F*_p1_.

The above experimental results clearly revealed that the Ti–6Al–4V surfaces were gently ablated at the *F*_p1_ between 1.1 to 2.9 J/cm^2^, while strongly ablated at the *F*_p1_ above 3.4 J/cm^2^. As mentioned in the Section 2.1, at the condition of LGA, soft physical processes of melting and re-solidifying occurred in the laser-material interface and thus flat surface with small roughness (*R*_a_ = 0.172–0.266 μm) was achieved at the *F*_p1_ between 1.1 to 2.9 J/cm^2^. However, as increasing *F*_p1_ to 3.4 J/cm^2^, because the applied laser fluence was above the SATF, violent physical processes occurred in the laser-material interface, resulting in the formation of distinctly sunk-raised microtextures. As consequence, sharp increase of surface roughness is observed at the *F*_p1_ of 3.4 J/cm^2^ in Figure 4a. The evolution law for the morphology and roughness of the Ti–6Al–4V surfaces with the increase of laser fluence was almost same as that for pure titanium (TA2) surfaces presented in our previous study. More details for the related mechanism analysis and discussion are available in reference [15]. However, it should be pointed out that at the same laser fluence, the roughness of the laser-irradiated Ti–6Al–4V surfaces were a little higher than that of the TA2 ones. This may be attributed to that the melting point of the metal alloys (Ti–6Al–4V) was generally lower than the corresponding pure metal (TA2), and thus more material was melted and ejected out, resulting in forming rougher surfaces.

#### 3.1.2. The Effect of Second-Step Laser Fluence

In this section, the effect of *F*_p2_ on the two-step laser-irradiated surface’ morphology was studied. Based on the results in the Section 3.1.1, in the two-step laser-irradiation process, the *F*_p1_ was selected to be above 3.4 J/cm^2^ for strongly ablating the Ti–6Al–4V surface, while the *F*_p2_ was employed in the range from 1.1 to 2.9 J/cm^2^ to perform gentle ablation. The scanning speed and hatching distance in both steps were applied to be constant of 42 mm/s and 1.0 μm, respectively.

**Figure 5:**
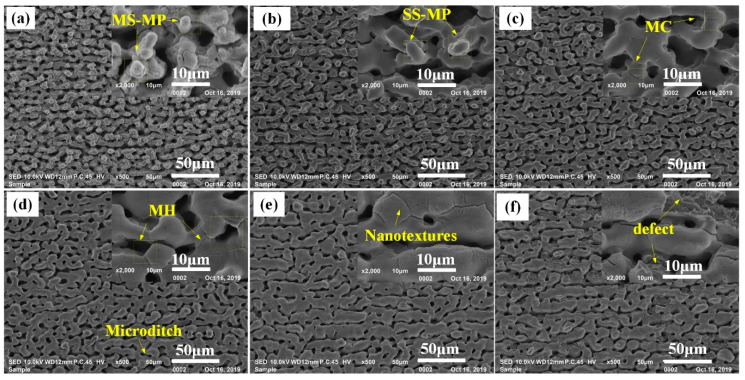
Morphology evolution of the surfaces irradiated by the two-step laser process with Fp1 = 4.0 J/cm^2^ and various *F*_p2_. (**a**) *F*_p2_ = 0.0 J/cm^2^; (**b**) *F*_p2_ = 1.1 J/cm^2^; (**c**) *F*_p2_ = 1.5 J/cm^2^; (**d**) *F*_p2_ = 1.9 J/cm^2^; (**e**) *F*_p2_ = 2.4 J/cm^2^; (**f**) *F*_p2_ = 2.9 J/cm^2^.

Figure 5 presents the morphology evolution of the Ti–6Al–4V surfaces irradiated by the two-step laser process with *F*_p1_ = 4.0 J/cm^2^ and various *F*_p2_. From Figure 5b, it could be clearly seen that the nanotextures initially covering on the top of microtextures in Figure 5a were successfully erased after performing the second-step laser process with the *F*_p2_ of 1.1 J/cm^2^. As a result, the remained microtexture surface was much smoother, which could be regarded as being single-scale. With increase of *F*_p2_ to 1.5 J/cm^2^ (Figure 5c), the surface of remained microtextures was still smooth except for forming some shallow MC like those in Figure 3, but the curvature of microtexture top obviously reduced. Further increasing *F*_p2_ ≥ 1.9 J/cm^2^, the surfaces filled with multi individual micro-holes/ditches were obtained. Note that, in Figure 5e, nanotextures were observed in some area of the laser-irradiated surface, like those in Figure 3d. Further increasing *F*_p2_ to 2.9 J/cm^2^, some defective regions were formed in the surface, as shown in Figure 5f. The morphology results of Figure 5 clearly shows that various types of microtextured Ti–6Al–4V surfaces (including multiscale microprotrusions (MS-MP), single-scale microprotrusions (SS-MP) as well as microholes (MH)) could be obtained through controlling the applied *F*_p2_.

**Figure 6:**
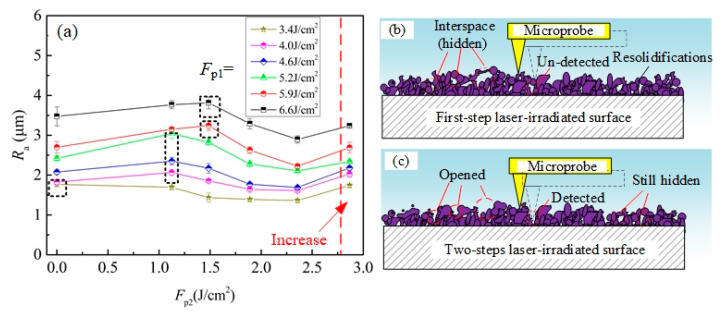
(**a**) Effect of *F*p2 on the roughness *R*a of two-step laser-irradiated surfaces; The illustration for roughness testing on the (**b**) first-step laser-irradiated and (**c**) two-step laser-irradiated surfaces.

**Figure 7:**
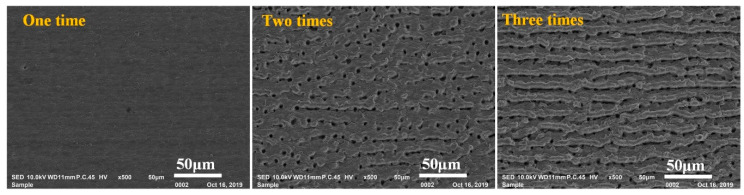
Effect of irradiating times on the morphology of Ti–6Al–4V surface at the laser fluence of 2.9 J/cm^2^.

Figure 6a presents the effect of *F*_p2_ on the roughness *R*a of the Ti–6Al–4V surfaces irradiated by two-step laser process. As seen from figure, the *R*a of the two-step laser-irradiated surfaces slightly increased first, and then gradually decreased with increasing *F*_p2_ from 0 to 2.4 J/cm^2^. It was important to note that the *F*_p2_ corresponding to the maximum *R*_a_ (the data in the black-dotted box in Figure 6a) presented an increasing trend with increasing *F*_p1_. For the two-step laser-irradiated surfaces using *F*_p1_ = 3.4 J/cm^2^, the *R*_a_ of them always decreased with increasing *F*_p2_ from 0 to 2.4 J/cm^2^, and thus the *F*_p2_ corresponding to the maximum *R*_a_ was 0.0 J/cm^2^. In addition, Figure 6a reveals that when the applied *F*_p2_ increased to 2.9 J/cm^2^, the roughness of the laser-irradiated surfaces increased again. Based on the mechanism of laser ablating solid material, when the target surface was strongly ablated by the laser, complex physics process occurred in the laser-material interface and multi molten materials were ejected out following by re-depositing and solidifying on the surface, resulting in the formation of raised microprotrusion textures. For laser-scanning irradiation process that line-by-line irradiation, when the applied hatching distance was small, the ejected melt derived from the later scanning lines would inevitably re-deposited on/between those derived from the previous scanning lines. As consequence, many hidden interspaces may exist between the overlapped re-solidifications. When applied roughness testing on this surface, these hidden interspaces could not be effectively detected, as shown in Figure 6b. However, after applying the second-step laser process, a layer of material on the top of microtextures was melted the thickness of the melt layer (*L*) can be determined by the following expression [20]:

(2) $$L=\alpha^{-1}\;\ln\;\left(F_{p}/F_{\mathrm{thg}}\right)$$

where *α* again is the absorption coefficient, *F*_thg_ is the gentle ablation-threshold fluence. Thus, after applied the second-step laser process with low *F*_p2_, the materials on the top of some interspaces were melt and the molten material trended to redistribute around the area adjacent to each initial surface asperity under the multidirectional action of surface tension [21]. As a result, the top of these interspaces would be opened, which showed as the increase of microholes/ditches in the surface. At the condition, when roughness testing was applied, the microprobe could successfully enter into these opened inner-spaces, as shown in Figure 6c. Therefore, though the nanotextures were successfully erased and the top of microprotrusion textures became flatter, the surface roughness still increased rather than decreased. When the *F*_p2_ was high enough to make all of the hidden interspaces exposed, the surface roughness reached to its maximum value. Further increasing the *F*_p2_, due to the increase of melting layer thickness, the surface roughness began to decrease. As seen from Figure 5, the amount of micro holes/ditches gradually increased with increase of *F*_p2_ first, and then had no distinct change with further increase *F*_p2_. This was agreed with the above analysis. During the first-step laser process, as higher *F*_p1_ was applied, more melt would be ejected out, and thus the formed re-solidification layer was thicker and more hidden interspaces existed in it. As consequence, the *F*_p2_ corresponding to the maximum *R*_a_ in Figure 6a presents an increasing trend with increasing *F*_p1_. At the *F*_p1_ of 3.4 J/cm^2^, because there were almost no hidden interspaces between the microtextures as shown in Figure 3e, the surface roughness always decreased with increase of *F*_p2_ from 1.1 to 2.4 J/cm^2^, and thus the *F*_p2_ corresponding to the maximum *R*_a_ was 0.0 J/cm^2^. According to the result presented in Figure 3d, though *F*_p1_ of 2.9 J/cm^2^ was quite close to the SATF of Ti–6Al–4V material, the target surface was still not strongly ablated, because almost no damage region caused by strong ablation was found in the laser-irradiated surface. However, for the two-step laser-irradiated surface, at the *F*_p2_ of 2.9 J/cm^2^ (Figure 5f), obvious strong ablation damage was observed in some regions, which lead to the increase of surface roughness again. This phenomena may be attributed to the incubation effect for the laser irradiation [21] that the SATF of Ti–6Al–4V material decreased with increase of the repeated irradiation times. In order to verify this description, the study for the effect of irradiation times on the surface morphology was performed at the *F*_p1_ of 2.9 J/cm^2^, and the related results are presented in Figure 7. As seen from figure, the surface was just gently ablated by one time irradiation at the laser fluence of 2.9 J/cm^2^, but strong ablation damage (microholes and shallow microridges) obviously formed as applying two and three times. These results were agreed with the above analysis.

### 3.2. Wettability and Surface Composition of the Laser-Irradiated Ti–6Al–4V surfaces

In this section, the wettability of the as-received, first-step laser-irradiated and two-step laser-irradiated Ti–6Al–4V surfaces before and after silanization were investigated. All the wetting tests were performed within two hours after the laser/silanization process. 

#### 3.2.1. Without Silanization

**Figure 8:**
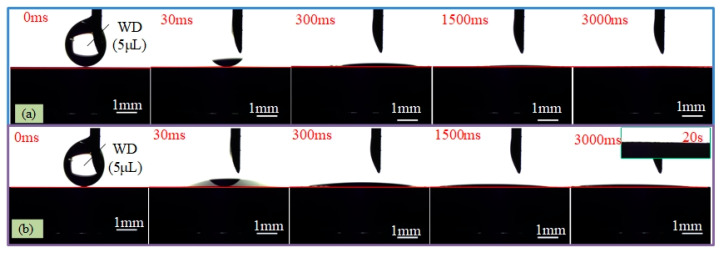
Sequential photographs of WCA measurement on (**a**) the first-step laser-irradiated surface using *F*_p1_ = 4.0 J/cm^2^ and (**b**) two-step laser-irradiated surface using *F*_p1_ = 4.0 J/cm^2^ along with *F*_p2_ = 2.4 J/cm^2^.

Figure 4b presents the WCAs of as-received (*F*_p1_ = 0 J/cm^2^) and first-step laser-irradiated surfaces at various *F*_p1_. It revealed that the as-received Ti–6Al–4V surface initially exhibited hydrophilicity with a WCA of 63.1 ± 2.4° and the hydrophilicity of it was clearly improved after the first-step laser process, manifested as the quick decrease of WCA with increasing *F*_p1_. When the applied *F*_p1_ was above 2.4 J/cm^2^, superhydrophilic surfaces with the WCA of less than 5° were obtained. While, all the two-step laser-irradiated surfaces, the processing parameters of which were same as those used in Figure 6a, exhibited superhydrophilicity with the WCA of nearly 0°. However, the time for the water droplet (WD) spreading on the two-step laser-irradiated surfaces obtained using the *F*_p2_ ≥ 2.4 J/cm^2^ to be steady state was a little longer than on those using *F*_p2_ between 1.1 and 1.9 J/cm^2^, especially at the end of the spreading process, as shown in Figure 8.

**Figure 9:**
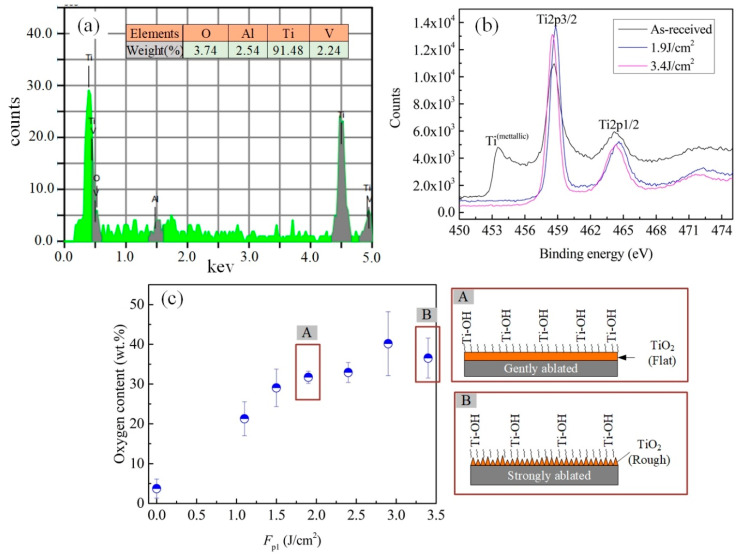
(**a**) EDS spectra of the as-received Ti–6Al–4V surface; (**b**) high-resolution XPS spectra (Ti2p) of the as-received and laser-irradiated Ti–6Al–4V surfaces; (**c**) effect of *F*_p1_ on the oxygen content in the laser-irradiated surfaces detected by EDS.

It is well known that the wettability of a solid surface is mainly determined by the two factors of chemical composition and roughness. According to the above results, it was believed that the WDs on the as-received and laser-irradiated Ti–6Al–4V surfaces were in Wenzel state. In this state, homogenous wetting occurs in the liquid–solid interface that the liquid could effectively penetrate into the valleys of the micro/nano-textures (illustration in Figure 4b), and the measured WCA (*θ*_r_) can be calculated by references [16,22]

(3) $$\cos\theta_{r}=r\cos\theta$$

where *θ* is the intrinsic contact angle on a smooth surface that relates to the material surface energy or composition, and *r* is the roughness factor that is defined as the ratio of the real solid–liquid contact area to the projected area. Based on the experimental results shown in Figure 4 and the principle of Wenzal model, it indicated that the enhancement of Ti–6Al–4V surface hydrophilicity irradiated at the *F*_p1_ ranging from 1.1 to 2.9 J/cm^2^ may be mainly attributed to the change of surface chemical composition rather than their roughness. This was because the increase of these surface roughness was so slight at the *F*_p1_ ranging from 1.1 to 2.9 J/cm^2^, which could not make the WCA decrease with such a large extent from 63.1 to 0° according to Equation (3). Figure 9 presents the EDS and XPS results of the as-received and some of the first-step laser-irradiated Ti–6Al–4V surfaces. All of these tests were also performed within two hours after the laser process. The EDS survey spectra in Figure 9a indicated that the as-received surface was slightly oxidized with the oxygen content of 3.74 wt.%. The high-resolution XPS spectra of the Ti2p core level (the black line in Figure 9b) showed the presence of a strong doublet with broad shoulders at the binding energies of 458.6 eV (Ti2p3/2), 464.4 eV (Ti2p1/2), which were attributed to Ti^4+^ [23,24]. In addition, note that, the spectrum characteristic Ti^Metallic^ (peaks at 453.5 eV) [25,26] was also observed in the XPS spectra of the as-received surface. This may be because the spontaneously formed oxide layer was quite thin, the underlying Ti–6Al–4V substrate was detected [26]. Based on the EDS and XPS results in Figure 9a,b, it revealed that a thin TiO_2_ film was spontaneously formed on the as-received Ti–6Al–4V surface. Due to the TiO_2_ was high-surface-energy material that could easily capture the -OH groups on water molecules, and thus the as-received surface was hydrophilic [19]. After performing the first-step laser process, the oxygen content in the Ti–6Al–4V surfaces measured by EDS (Figure 9c) gradually increased with increase of *F*_p1_, which indicated more titanium oxides formed on the surface. In addition, according to the XPS spectra of the laser-irradiated surfaces (the pink and blue lines in Figure 9b), these titanium oxides were still mainly consisted of TiO_2_. However, different from that of the as-received one, the spectrum characteristic of Ti^Metallic^ was not observed in the laser-irradiated surfaces. This was because the TiO_2_ film on the laser-irradiated surfaces was thick enough, and the underlying Ti–6Al–4V substrate was not detected by XPS [18]. The increase of TiO_2_ content were quite beneficial for the enhancement of hydrophilicity. Therefore, the Ti–6Al–4V surface hydrophilicity was improved after laser irradiation using *F*_p1_ ranging from 1.1 to 2.9 J/cm^2^, though the roughness of them was so close to each other. In the reference [19], for the laser-irradiated surface with the WCA of about 30–35°, the surface roughness *R*a of it reached to be as much as 2.0–2.5 μm, which was about ten times to the one with almost the same WCA in this paper (The data points in Figure 4b at the *F*_p1_ = 1.1 J/cm^2^, *R*a = 0.204 ± 0.011 μm and WCA = 31.3 ± 1.0°). This may because in the reference [19] the laser process was performed in a vacuum chamber and almost no more titanium oxides would form on the surface after laser irradiation. As a result, it needed to get much rougher surface for enhancing the hydrophilicity, according to the Wenzel model. For the two-step laser-irradiated surfaces, not only the high TiO_2_ content, but also the high roughness were beneficial for enhancing the hydrophilicity of them, and thus all of them exhibited superhydrophilicity. However, for the surfaces with multi individual microholes obtained at the *F*_p2_ above 2.4 J/cm^2^, though the water could effectively penetrate into these microholes finally, the air trapped in the bottom of microholes still slowed down the speed of water penetration to some extent. Therefore, the time for the WDs completely spreading on them was a little longer than that on the surfaces with microprotrusion textures.

#### 3.2.2. With Silanization

**Figure 10:**
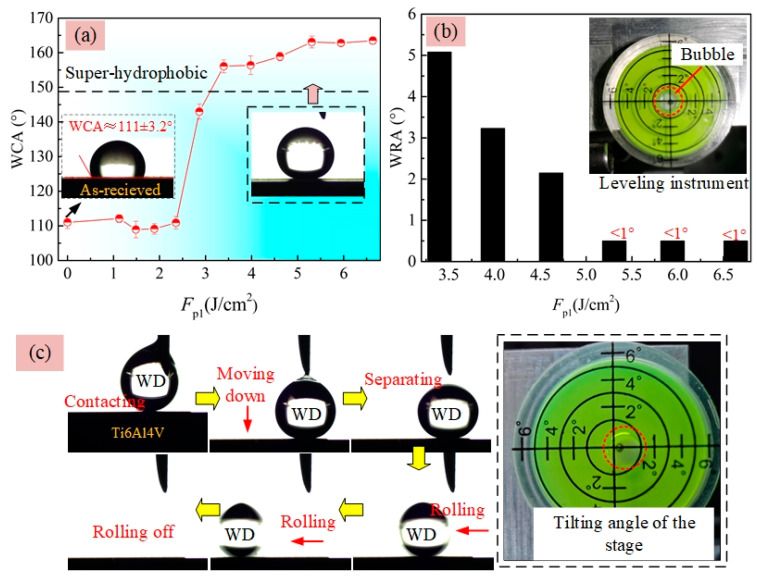
Effect of *F*_p1_ on the (**a**) WCA and (**b**) WRA of Ti–6Al–4V surfaces produced by first-step laser irradiation following with silanization; (**c**) sequential photographs of WCA measurement on the superhydrophobic surface obtained at the *F*_p1_ of 5.2 J/cm^2^ as tilting the stage with small angle of less than 1°.

**Figure 11:**
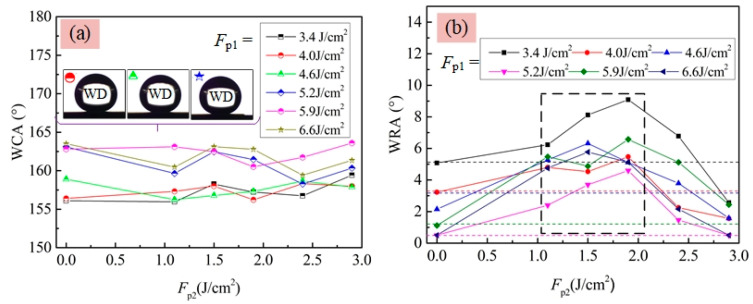
Effect of *F*_p2_ on the (**a**) WCA and (**b**) WRA of two-step laser-irradiated Ti–6Al–4V surfaces with silanization.

After silanization, all the initial hydrophilic Ti–6Al–4V surfaces presented in the Section 3.2.1, including the as-received, first-step and two-step laser-irradiated surfaces, turned to be hydrophobic with the WCA above 90° and even superhydrophobic with the WCA above 150°. This was because the surface energy of the Ti–6Al–4V samples were effectively reduced by the FAS. Note that, the laser irradiation parameters of the first-step laser-irradiated samples used in Figure 10 were same as those in Figure 4, and the two-step laser-irradiated samples used in Figure 11 were same as those in Figure 6a.

Figure 10a shows that the WCAs of the hydrophobic surfaces obtained at the *F*_p1_ ranging 0 to 2.4 J/cm^2^ with silanization were close to each other (109–112°). At the *F*_p1_ of 2.9 J/cm^2^, distinct increase of WCA to be 143° was observed though the surface roughness (*R*_a_ = 0.266 μm)) was still relatively small according to Figure 4a. Further increasing *F*_p1_, superhydrophobic surfaces with the WCA of 156–163° were achieved. For a superhydrophobic surface, the WRA is another important factor for characterizing their wettability, which can be used to reflect the adhesion property in the liquid–solid interface. Here, the WRA on those superhydrophobic Ti–6Al–4V surfaces in Figure 10a were also measured. Figure 10b clearly shows that the adhesion forces between WDs and the superhydrophobic surfaces were quite small because the WRAs of them were less than 5°. Note that, before performing the WRA tests, the stage used to hold the substrate was adjusted to be horizontal as much as possible, assisted by using a simple leveling instrument as shown in the inset figure of Figure 10b. In addition, from Figure 10b, it could be seen that the WRA of superhydrophobic surfaces presented a decrease trend with increasing *F*_p1_ from 3.4 to 6.6 J/cm^2^. When the laser-irradiated surfaces obtained using *F*_p1_ ≥ 5.2 J/cm^2^ were just titled with an angle of less than 1° in advance, the WDs could spontaneously rolled off from them, once separating with the syringe needle, as shown in Figure 10c. This indicated the adhesion force between the WD and the superhydrophobic Ti–6Al–4V surface was quite small. Figure 11 presents the WCA and WRA results of the two-step laser-irradiated surfaces after silanization. From Figure 11a, it could be seen that all of two-step laser-irradiated surfaces turned to be superhydrophobic with the WCAs in the range from 155° to 162° after silanization, but no obvious evolution law of the WCA with the increase of *F*_p2_ were observed. However—compared with those of the first-step laser-irradiated ones—the WRAs of the Ti–6Al–4V surfaces clearly increased after applying the second-step laser-irradiation process using *F*_p2_ ranging from 1.1 to 1.9 J/cm^2^ though the increments were not much, as shown in Figure 11b.

**Figure 12:**
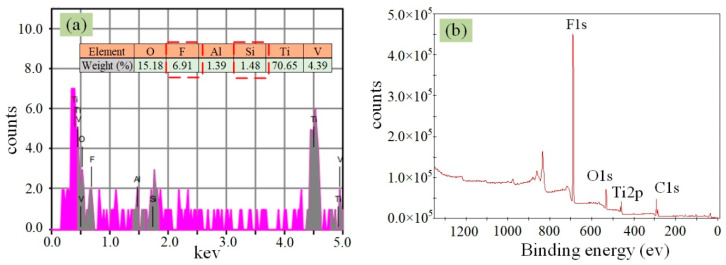
(**a**) EDS and (**b**) XPS spectra of the Ti–6Al–4V surface after silanization.

According to previous studies, a self-assembled FAS molecular film will form on the Ti–6Al–4V surface after silanization process, due to the fact that hydrolyzed FAS molecules reacting with the –OH groups on the surface and crosslinking among adjacent hydrolyzed FAS molecules [11]. The FAS film is low surface energy material, and thus it can easily make an initial hydrophilic surface turn to be hydrophobic. Figure 12 presents the EDS and XPS spectra of the Ti–6Al–4V surface with silanization. As seen from the figure, the Si and F elements with 6.91 wt.% and 1.48 wt.% respectively were clearly detected by using EDS, and high peak value of F1 s was observed in the XPS spectrum. This indicated FAS molecular film successfully formed on the Ti–6Al–4V surface after silanization process [5,9,27]. For the laser-irradiated Ti–6Al–4V surfaces with silanization, their chemical composition was same (FAS). Therefore, there was no distinct change of WCAs on the first-step laser-irradiated surfaces using *F*_p1_ ranging from 1.1 to 2.4 J/cm^2^ after silanization, because the surface roughness of them were close to each other (Figure 4a). However, at the *F*_p1_ of 2.9 J/cm^2^, distinct increase of WCA to be 143° was observed though the surface roughness (*R*a = 0.266 μm)) was still relatively small. This may because the formation of nanotextures on the laser-irradiated Ti–6Al–4V surface using *F*_p1_ of 2.9 J/cm^2^ sustained the air pressure within the valleys of them, which prevented the water spreading and permeating. Figure 10b shows that the WDs on those superhydrophobic surfaces were in heterogeneous wetting regime (so-called Cassie state), because only in the Cassie state does a hydrophobic surface exhibit a small WRA (typically smaller than 10°) [19]. The mechanism of Cassie state has been well discussed in many previous papers. In the Cassie state, liquid did not penetrate into, but rather suspend on the microtextures, because the air was trapped in the spaces between them. As a result, the WD could easily roll off from the solid surface. After applying the second-step laser process using *F*_p2_ ranging from 1.1 to 1.9 J/cm^2^, the nanostructures were absent in the laser-irradiated surfaces, and thus the effective contacting area between the WD and the solid surface enlarged. This may be attributed to the increase of WRA. At the *F*_p2_ above 2.4 J/cm^2^, nanotextures were formed on the surfaces again, and thus the WRA decreased. These results indicated the micron-rough surface with single-scale microtextures were feasible to achieve superhydrophobicity with relative high adhesion energy.

#### 3.2.3. Multifunctional Superhydrophobic Surfaces Produced by Two-Step Laser-Irradiation Process

**Figure 13:**
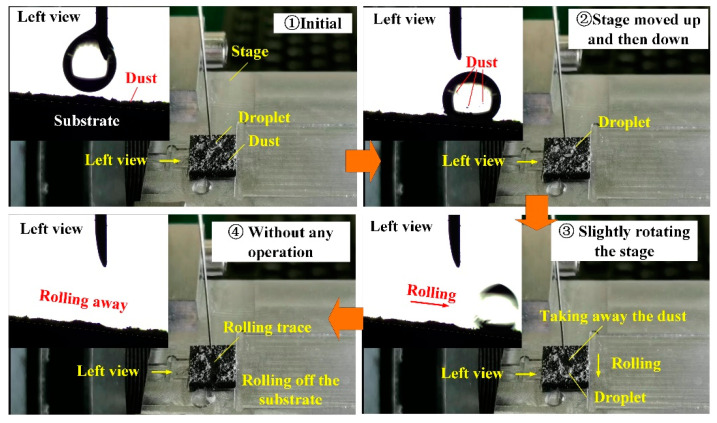
Functional surface with self-cleaning property.

**Figure 14:**
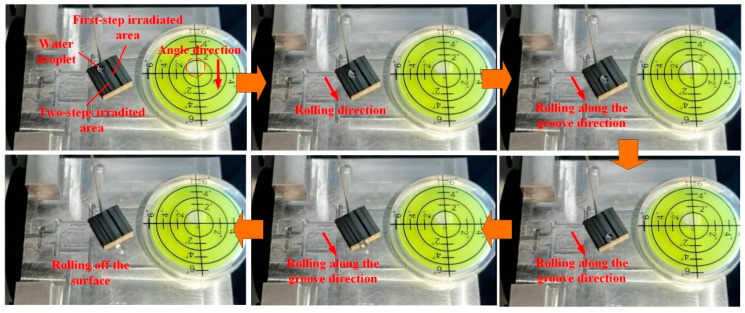
Functional surface with guiding property produced by using *F*_p1_ = 5.2 J/cm^2^ and *F*_p2_ = 2.9 J/cm^2^.

**Figure 15:**
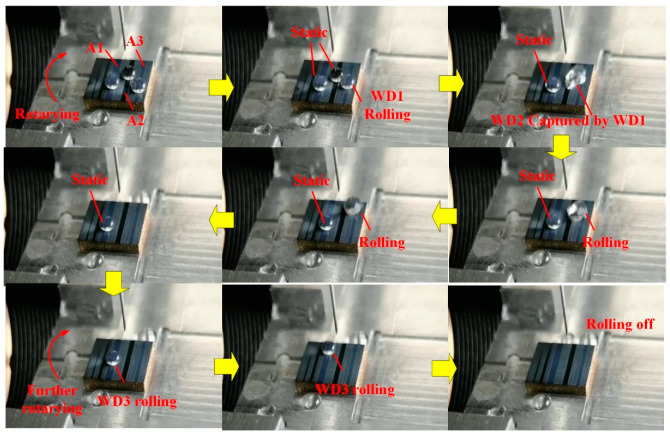
Superhydrophobic surface with different adhesion forces to water droplet produced by using *F*_p1_ = 6.6 J/cm^2^ and various *F*_p2_ of A1 = 1.5 J/cm^2^, A2 = 1.9 J/cm^2^ and A3 = 2.4 J/cm^2^.

Figure 13 and video S1 in Supplementary Materials clearly show that the micron-rough surfaces with single-scale microtextures obtained by using the two-step laser-irradiated method with *F*_p1_ = 4.0 J/cm^2^ and *F*_p2_ = 1.5 J/cm^2^ also owned the self-cleaning property that the water droplet could easily roll off the surface and take away the dusts. Figure 14 and video S2 in Supplementary Materials indicate that the two-step laser-irradiation method could be used to produce the superhydrophobic surfaces with the guiding property just by applying the second-step laser process in a selective region, which has a potential application in the microfluidic field. Figure 15 and video S3 in the Supplementary Materials clearly show that the adhesion energy of the surface could be controlled to be different in selective regions by adjusting the value of the applied *F*_p2_, resulting in able to select a certain water droplet to roll off at some point. In addition, it indicated that the guiding property produced by the two-step laser method could also undergoes the lateral force to some extent that the first water droplet rolled and captured and emerged with the second droplet on the side, but almost still rolled along the initial direction after this.

**Figure 16:**
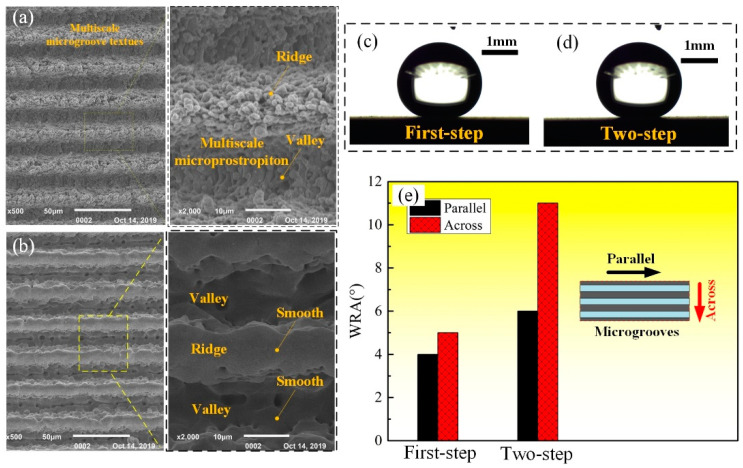
SEM morphology of microgroove textures obtained by (**a**) first-step and (**b**) two-step laser-scanning irradiation with the parameters listed in Table 2; Photographs for WCA measurement of the anterior-mentioned (**c**) first-step and (**d**) two-step laser-irradiated surfaces after silanization; (**e**) WRAs along the directions parallel to and across the microgrooves of the first and single-scale microgroove textures.

**Figure 17:**
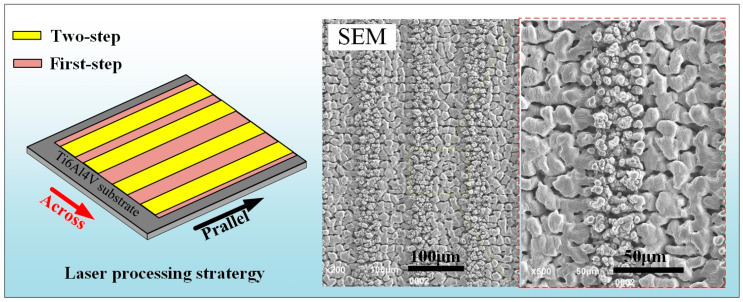
Functional surface with groove-like hybrid multi- and single- scale microtextures produced by using two-step laser irradiation with *F*_p1_ = 4.0 J/cm^2^ and *F*_p2_ = 2.4 J/cm^2^.

This method was also effective to be applied in erasing the nanostructures on the multiscale microgroove textures produced by using the method presented in our previous work (Figure 16a) [15] resulting in the production of microgroove textures with smooth surfaces (Figure 16b). After silanization, both of the multiscale and single-scale microgroove textures exhibited superhydrophobicity with the WCA of 162.3° (Figure 16c) and 160.2° (Figure 16d). However, note that, compared with the multiscale ones, the single-scale microgroove textures not only owned larger WRA, but the anisotropy of it was more distinct, as shown in Figure 16e. The anisotropic dewetting tendency is also a quite popular property for achieving functional surface and have recently attracted much interest [28]. Moreover, functional surface with groove-like hybrid multi- and single- scale microtextures were successfully produced on the Ti–6Al–4V surface, as shown in Figure 17. After silanization, this surface also exhibited superhydrophobicity with a WCA of about 158.4° and slight anisotropic WRAs on it were also observed that the WRA along the parallel direction was about 3.2° and the one along the across direction was about 4.9°.

## 4. Conclusions

In summary, a method of two-step laser-scanning irradiation coupling successive strong ablation and gentle ablation was presented for obtaining micron-rough surface with single-scale microtextures. The scope of laser fluence for gentle ablation of Ti–6Al–4V surfaces were found to be in the range from 1.1 to 2.9 J/cm^2^ and that for strong ablation to be above 3.4 J/cm^2^ at the scanning speed of 42 mm/s and hatching distance of 1.0 μm. Applying the laser fluence above 3.4 J/cm^2^, micron-rough surfaces with multiscale microprotrusion textures were obtained, the *R*_a_ and *R*_z_ of which were above 1.767 μm and above 11.199 μm, respectively. When applying the second-step laser process with the laser fluence 1.1 to 1.9 J/cm^2^, the nanotextures could be successfully erased and micron-rough surface with single-scale microtextures achieved. Due to existing of hidden interspaces between the microprotrusion textures obtained by the first-step laser process, the surface roughness increased first, and then decreased with increase of *F*_p2_ from 1.1 to 2.9 J/cm^2^ after applying the second-step laser process. All of these surfaces irradiated by the two-step laser process exhibited superhydrophilicity though the morphologies of the microtextures on them were different and turned to be superhydrophobic after silanization. The adhesion energy of the obtained superhydrophobic surfaces could be enhanced by applying *F*_2p_ in the range from 1.1 to 1.9 J/cm^2^, due to the absence of nanotextures on the micron-rough surfaces. Moreover, it was confirmed that through applying the second-step laser process in the selective regions, superhydrophobic surfaces with multi functions could be achieved, including self-cleaning, relative high adhesion with large water contact angle, guiding of the water-rolling direction as well as the superhydrophobic surface with anisotropic water-rolling angle like the rice-leaf, etc. The results imply the potential applications of these surfaces in a variety of fields, such as biomedical, microfluidic, etc.

## Figures and Tables

**Table 1:** Nominal composition of the used Ti–6Al–4V material.

Elements	Ti	Al	V	Fe	C	O	H	N
Weight%	Balance	5.5–6.8	3.5–4.5	≤0.3	≤0.1	≤0.2	≤0.015	≤0.05

**Table 2:** Laser parameters applied for fabricating the microgroove textures shown in Figure 16.

	*F*_p_ (J/cm^2^)	*v* (mm/s)	*h* (μm)
First-step	13.2	42.0	30.0
Second-step	1.9	42.0	1.0

## Supplementary Materials

The following are available online at https://www.mdpi.com/1996-1944/13/17/3816/s1, Video S1: video 1.mp4, Video S2: video 2.mp4, Video S3: video 3.mp4.

## References

[1] Song Y., Liu Y., Zhan B., Kaya C., Stegmaier T., Han Z.W., Ren L.Q. (2017). Fabrication of bioinspired structured superhydrophobic and superoleophilic copper mesh for efficient oil-water separation. J. Bion. Eng..

[2] Trdan U., Hocevar M., Gregorcic P. (2017). Transition from superhydrophilic to superhydrophobic state of laser textured stainless steel surface and its effect on corrosion resistance. Corros. Sci..

[3] Bhushan B., Jung Y.C. (2011). Natural and biomimetic artificial surfaces for superhydrophobicity, self-cleanning, low adhesion, and drag reduction. Progress Mater. Sci..

[4] Yang Y., Lai Y.K., Zhang Q.Q. (2010). A novel electrochemical strategy for improving blood compatibility of titanium-based biomaterials. Colloids Surf. B Biointerfaces.

[5] Chen L., Han D., Jiang L. (2011). On improving blood compatibility: From bioinspired to synthetic design and fabrication of biointerfacial topography at micro/nano scales. Colloids Surf. B Biointerfaces.

[6] Doll K., Fadeeva E., Stumpp N.S., Grade S., Chichkov B.N., Stiesch M. (2016). Reduced bacterial adhesion on titanium surfaces micro-structured by ultra-short pulsed laser ablation. BioNanoMaterials.

[7] Wu B., Zhou M., Li J., Ye X., Li G., Cai L. (2009). Superhydrophobic surfaces fabricated by microstructuring of stainless steel using a femtosecond laser. Appl. Surf. Sci..

[8] Liu W.J., Cai M.Y., Luo X., Chen C.H., Pan R., Zhong H.J., Zhong M.L. (2019). Wettability transition modes of aluminum surfaces with various micro/nanostructures produced by a femtosecond laser. J. Laser Appl..

[9] Long J.Y., Cao Z., Lin C.H., Zhou C.X., He Z.J., Xie X.Z. (2019). Formation mechanism of hierarchical Micro- and nanostructures on copper induced by low-cost nanosecond lasers. Appl. Surf. Sci..

[10] Li B.J., Zhou M., Yuan R., Cai L. (2008). Fabrication of titanium-based microstructured surfaces and study on their superhydrophobic stability. Mater. Res. Soc..

[11] Chen T.C., Liu H.T., Yang H.F., Yan W., Zhu W., Liu H. (2016). Biomimetic fabrication of robust self-assembly superhydrophobic surfaces with corrosion resistance properties on stainless steel substrate. RSC Adv..

[12] Yang Z., Liu X.P., Tian Y.L. (2019). Hybrid laser ablation and chemical modification for fast fabrication of bio-inspired super-hydrophobic surface with excellent self-cleanning, stability and corrosion resistance. J. Bion. Eng..

[13] Fadeeva E., Truong V.K., Stiesch M., Chichkov B.N., Crawford R.J., Wang J., Ivanova E.P. (2011). Bacterial retention on superhydrophobic titanium surfaces fabricated by femtosecond laser ablation. Langmuir.

[14] Xin G.Q., Wu C.Y., Cao H.Y., Liu W.N., Li B., Huang Y., Rong Y.M., Zhang G.J. (2020). Superhydrophobic TC4 alloy surface fabricated by laser micro-scanning to reduce adhesion and drag resistance. Surf. Coat. Technol..

[15] He H.D., Wang C.J., Zhang X., Ning X.Z., Sun L.N. (2020). Facile fabrication of multi-scale microgroove textures on Ti-based surface by coupling the re-solidification bulges derived from nanosecond laser irradiation. Surf. Coat. Technol..

[16] He H.D., Qu N.S., Zeng Y.B. (2016). Lotus-leaf-like microstructures on tungsten surface induced by one-step nanosecond laser irradiation. Surf. Coat. Technol..

[17] Bordatchev E.V., Hafiz A.M.K., Tutunea-Fatan O.R. (2014). Performance of laser polishing in finishing of metallic surfaces. Int. J. Adv. Manufact. Technol..

[18] Cunha A., Serro A.P., Oliveira V., Almeida A. (2013). Wetting behavior of femtosecond laser textured Ti-6Al-4V surfaces. Appl. Surf. Sci..

[19] Li B.J., Li H., Huang L.J., Ren N.F., Kong X. (2016). Femtosecond pulsed laser textured titanium surfaces with stable superhydrophilicity and superhydrophobicity. Appl. Surf. Sci..

[20] Cheng J., Liu C.S., Shang S., Liu D., Perrie W., Dearden G., Watkins K. (2013). A review of ultrafast laser materials micromachining. Opt. Laser Technol..

[21] Liang J.C., Liu W.D., Li Y., Luo Z., Pang D.Q. (2018). A model to predict the ablation width and calculate the ablation threshold of femtosecond laser. Appl. Surf. Sci..

[22] Gregorcic P., Setina-batic B., Hocevar M. (2017). Controlling the stainless steel surface wettability by nanosecond direct laser texturing at high fluences. Appl. Phys. A.

[23] Skowronski L., Antonczak A.J., Trzcinski M., Lazarek L., Hiller T., Bukaluk A., Wronkowska A.A. (2014). Optical properties of laser induced oxynitride films on titanium. Appl. Surf. Sci..

[24] Akman E., Cerkezoglu E. (2016). Compositional and micro-scratch analyses of laser induced colored surface of titanium. Opt. Lasers Eng..

[25] Antonczak A.J., Skowronski L., Trzcinski M., Kinzhybalo V.V., Lazarek L.K., Abramski K.M. (2015). Laser-induced oxidation of titanium substrate: Analysis of the physicochemical structure of the surface and sub-surface layers. Appl. Surf. Sci..

[26] Lee M.H., Oh N., Lee S.W., Leesungbok R., Kim S.E., Yun Y.P., Kang J.H. (2010). Factors influencing osteoblast maturation on microgrooved titanium substrata. Biomaterials.

[27] Lu Y., Xu W.J., Song J.L., Liu X., Xing Y.J., Sun J. (2012). Preparation of superhydrophobic titanium surfaces via electrochemical etching and fluorosilane modification. Appl. Surf. Sci..

[28] Yu H.D., Lian Z.X., Wan Y.L., Weng Z.K., Xu J.K., Yu Z.L. (2015). Fabrication of durable superamphiphobic aluminum alloy surfaces with anisotropic sliding by HS-WEDM and solution immersion processes. Surf. Coat. Technol..

